# A case report of HER2-positive descending colon cancer with peritoneal metastasis and literature review

**DOI:** 10.3389/fonc.2025.1473620

**Published:** 2025-03-14

**Authors:** Yaochun Lv, Zhengpeng Qian, Dewang Wu, Chengzhang Zhu, Jipeng Zhang, Yeping Ning, Binbin Du

**Affiliations:** ^1^ Gansu Clinical Medical Research Center for Anorectal Diseases, Lanzhou, Gansu, China; ^2^ The First Clinical Medical College, Gansu University of Chinese Medicine, Lanzhou, Gansu, China; ^3^ Clinical Medical Research Center for Anorectal Diseases of Gansu Province, Gansu Provincial Hospital, Lanzhou, China; ^4^ The First Clinical Medical College of Lanzhou University, Lanzhou, Gansu, China

**Keywords:** HER2-positive descending colon cancer, peritoneal metastasis, anti-HER2 therapy, multidisciplinary collaboration (MDT), case report

## Abstract

Human epidermal growth factor receptor 2 (HER2) is an anti-cancer drug target for colon cancer. Among patients with colorectal malignancy (colorectal cancer, CRC), those with HER2 mutations have a poor overall prognosis and a significantly increased drug resistance. In recent years, anti-HER2 therapeutic drugs have developed rapidly. According to several clinical studies and case reports, anti-HER2 therapy, as an emerging anti-cancer approach, plays a crucial role in the treatment of HER2-positive CRC patients. Here, we present a case of HER2-positive descending colon cancer with peritoneal metastasis. The patient is a 26-year-old male, diagnosed with malignant tumor of the descending colon with peritoneal metastasis in April 2020. After multiple treatment modalities, the disease progressed. After chemotherapy with Trastuzumab Deruxtecan (T-DXd/DS-8201), the metastatic foci significantly shrank, and after surgical resection, a tumor-free state (NED) was achieved. Up to now, the patient’s survival period has reached 56 months.

## Introduction

CRC represents a prevalent malignant tumor within the gastrointestinal tract. Regrettably, a substantial portion of CRC cases are already in advanced stages upon detection. Presently, it holds the third position in terms of global incidence and the second position in mortality rates (1).

The treatment of early and mid-stage CRC mainly includes surgical resection, chemotherapy, radiotherapy, and immunotherapy, etc. The prognosis of the colorectal cancer (CRC) population is generally unfavorable. Treatment strategies are formulated based on specific biomarkers and clinical stratification, thereby determining the most appropriate regimens such as radiotherapy, chemotherapy, immunotherapy, and surgery in accordance with the individualized characteristics of patients (2). Tumor metastasis is a fatal factor for advanced CRC. Approximately 22% of CRC cases have metastasis at the time of onset, and 19% of cases will develop metachronous metastasis (3). Among them, peritoneal metastasis is an important metastasis route of CRC. In addition to traditional systemic and surgical treatments, targeted therapies have also been incorporated into the diagnosis and treatment of advanced metastatic CRC. Although the diagnosis and treatment level of advanced CRC has been continuously improving in recent years, the prognosis of advanced CRC is often unsatisfactory compared with that of early and mid-stage CRC. Most metastatic colorectal cancer (mCRC) is difficult to cure, but with the development of chemotherapy and targeted drugs, the survival rate of these patients has increased. And the new targeted HER2 treatment may bring hope for these special patients (4, 5).

HER2 plays an important role in normal biology and the carcinogenic process, and regulates cell proliferation, differentiation and migration through a variety of signal transduction pathways, such as mitogen-activated protein kinase/extracellular signal-regulated kinase (MAPK/ERK) and phosphatidylinositol 3 kinase (PI3K)/Akt/mammalian target of rapamycin (mTOR), etc., to regulate cell functions (6). As a member of the receptor tyrosine kinase ERBB family, HER2 plays an important role in normal biology and tumorigenesis by driving multiple downstream signal transduction pathways, the most significant of which are the mitogen-activated protein kinase (MAPK) and phosphatidylinositol 3-kinase pathways. Abnormal activation of the MAPK pathway is one of the first key molecular alterations identified in CRC and usually represents an early step in tumorigenesis. Approximately 60% to 80% of colorectal adenocarcinomas have activating alterations in the MAPK pathway (7). In recent years, T-DXd has been gradually approved for application in a variety of HER2-positive solid tumors. T-DXd is a new type of anti-HER2 antibody-drug conjugate (ADC) composed of trastuzumab, a 4-peptide linker and an exatecan derivative. Studies have shown that many patients with HER2-positive solid tumors can benefit from T-DXd, and it has also shown significant effects in patients with HER2-positive CRC (8, 9).

Here, we report a patient with HER2-positive malignant tumor of the descending colon and peritoneal metastasis. At of the time of this manuscript submission, this patient has survived for 56 months. Currently, the patient is in a NED(No Evidence of Disease) state.

## Case report

The patient, a 26-year-old young man, was admitted to the hospital on April 10, 2020, mainly due to “intermittent abdominal distension for more than 6 months and the discovery of descending colon cancer for 8 days”. Six months before admission, he had intermittent abdominal distension with intermittent bloody stool without cause. The bloody stool was dark red without mucus or pus and improved after self-symptomatic treatment. On April 2, 2020, the patient’s anus stopped exhausting and defecating. He was diagnosed with “intestinal obstruction” when visiting an outside hospital. After the intestinal stent was placed by endoscopy in the emergency department, the obstructive symptoms improved. Colonoscopy showed: a mass in the descending colon. Pathological examination of the biopsy showed: moderately differentiated adenocarcinoma of the descending colon. Thus, he was admitted to the hospital with “malignant tumor of the descending colon”. Since the onset of the disease, the patient was conscious with a good spirit, poor diet, good sleep, and had a weight loss of about 3kgs in the past 3 months. He had no previous history of special diseases and his mother has a personal history of thyroid malignancy. BMI: 23.53kg/m2, Body surface area: 1.78m2. Physical examination: The vital signs are stable. There were no enlarged lymph nodes or pedal edema on clinical examination. The remainder of the systemic examination was unremarkable. During the per-rectal examination (extending up to 7 centimeters from the anal verge), dark red blood was observed on the gloved finger.

Auxiliary examinations revealed a CEA level of 2.01 ng/ml and a CA199 level of < 2.0 U/ml. Electronic colonoscopy demonstrated that upon insertion of the endoscope to the junction of the descending and sigmoid colon, a significant amount of pasty feces was observed. At this junction, a new growth encircling the wall was detected, and the endoscope could not progress further due to luminal stenosis. Pathological examination of the colonoscopy specimen indicated moderately differentiated adenocarcinoma at the junction of the descending and sigmoid colon. Thoracic and abdominal CT scans exhibited no conspicuous abnormalities within the chest. However, a tumorous transformation near the splenic flexure of the descending colon was discerned. Additionally, multiple metastatic foci were detected in the greater omentum and abdominopelvic cavity, corresponding to the classification of (pT4bN0M1c, stage IV).

Diagnosis: Descending colon cancer; Incomplete intestinal obstruction; Peritoneal metastasis of malignant tumor.

After admission, the surgical contraindications were excluded. On April 16, 2020, laparoscopic left hemicolectomy + cytoreductive surgery + colostomy were performed. During the operation, it was seen that the tumor was located near the splenic flexure of the descending colon, penetrating the serous layer and invading the adjacent peritoneum. One metastatic lesion about 3×2 cm in size was seen in the greater omentum, and multiple metastatic lesions of different sizes were seen on the peritoneal membranes in the abdominal and pelvic cavities (PCI=7).

**Figure 1:**
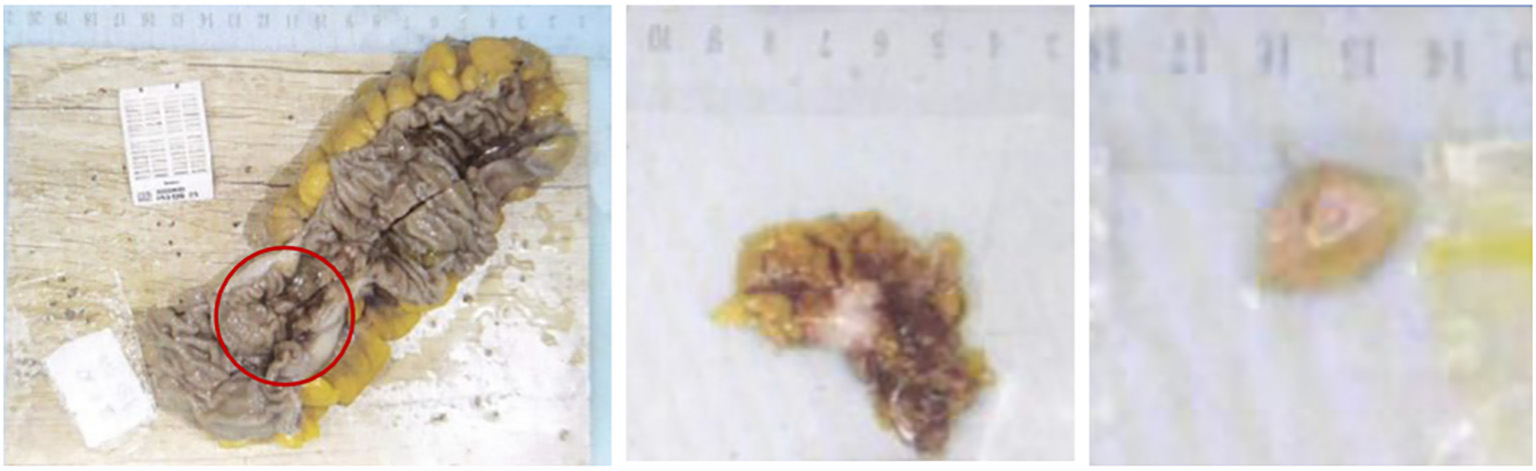
The resected left half of the colon and abdominal wall nodules.

**Figure 2:**
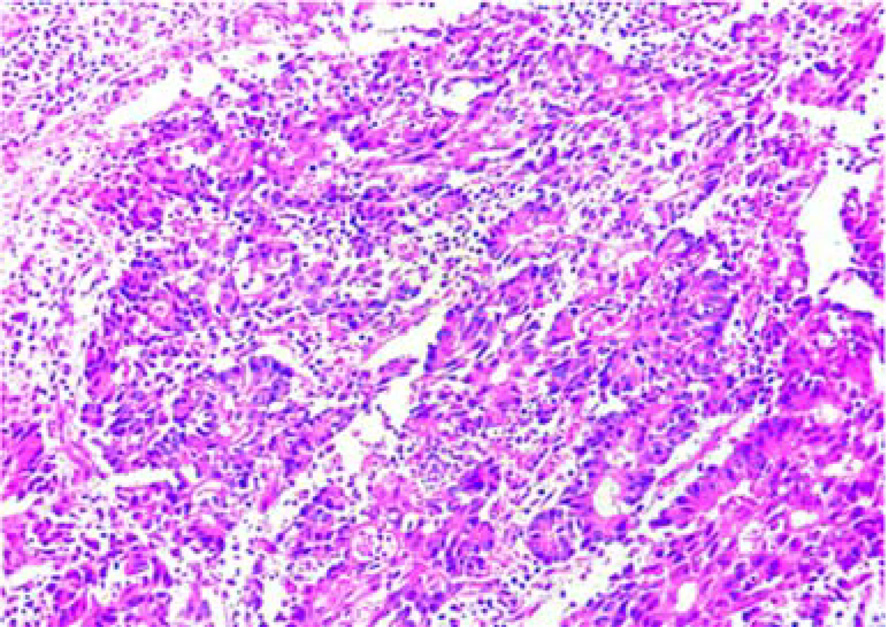
Pathological examination after the first surgery 10*20 HE .

**Figure 3:**
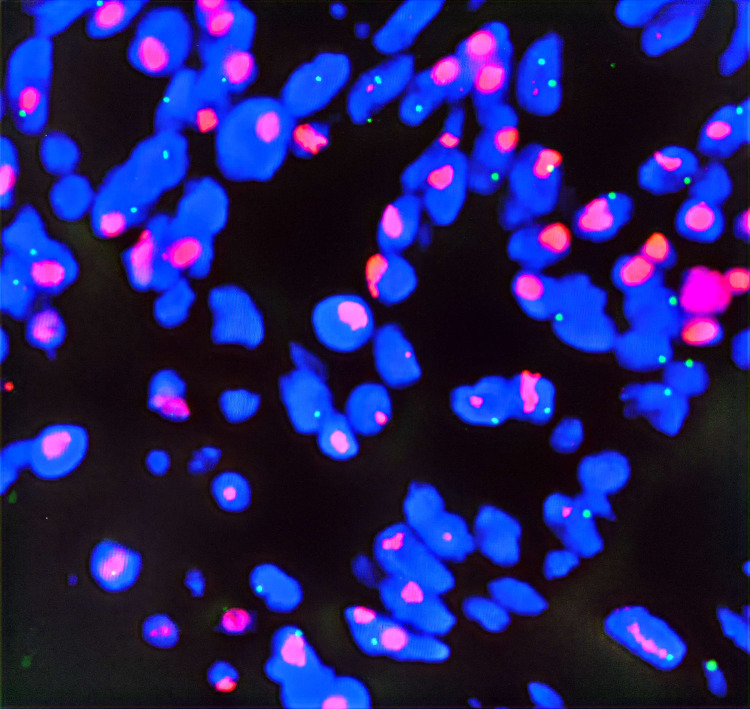
HER2 *in situ* hybridization photo.

Postoperative pathological diagnosis: Left-sided colon cancer, ulcerative type, moderately differentiated tubular adenocarcinoma. The cancer tissue penetrated the entire layer of the intestinal wall. Cancer thrombus was seen in the blood vessels. The cancer tissue invaded the nerves. No cancer tissue was involved in the upper and lower resection ends and the lateral resection margin. No metastatic cancer was found in the lymph nodes (0/13). Additionally sent “Omental nodule, peritoneal nodule 1 and abdominal wall nodule 2”: All the submitted tissues were cancer nodules (**Figures 1** and **2**). FISH Results: The HER2/CEP17 ratio was determined to be 3.5, and the average HER2 copy number was measured at 8.2. These findings unequivocally indicate that the HER2 gene was amplified, thereby suggesting that the patient may potentially be a candidate for anti-HER2 targeted therapy. Additionally, the immunohistochemistry result from our hospital demonstrated that the patient was HER2 positive (3+) (**Figure 3**).

**Figure 4:**
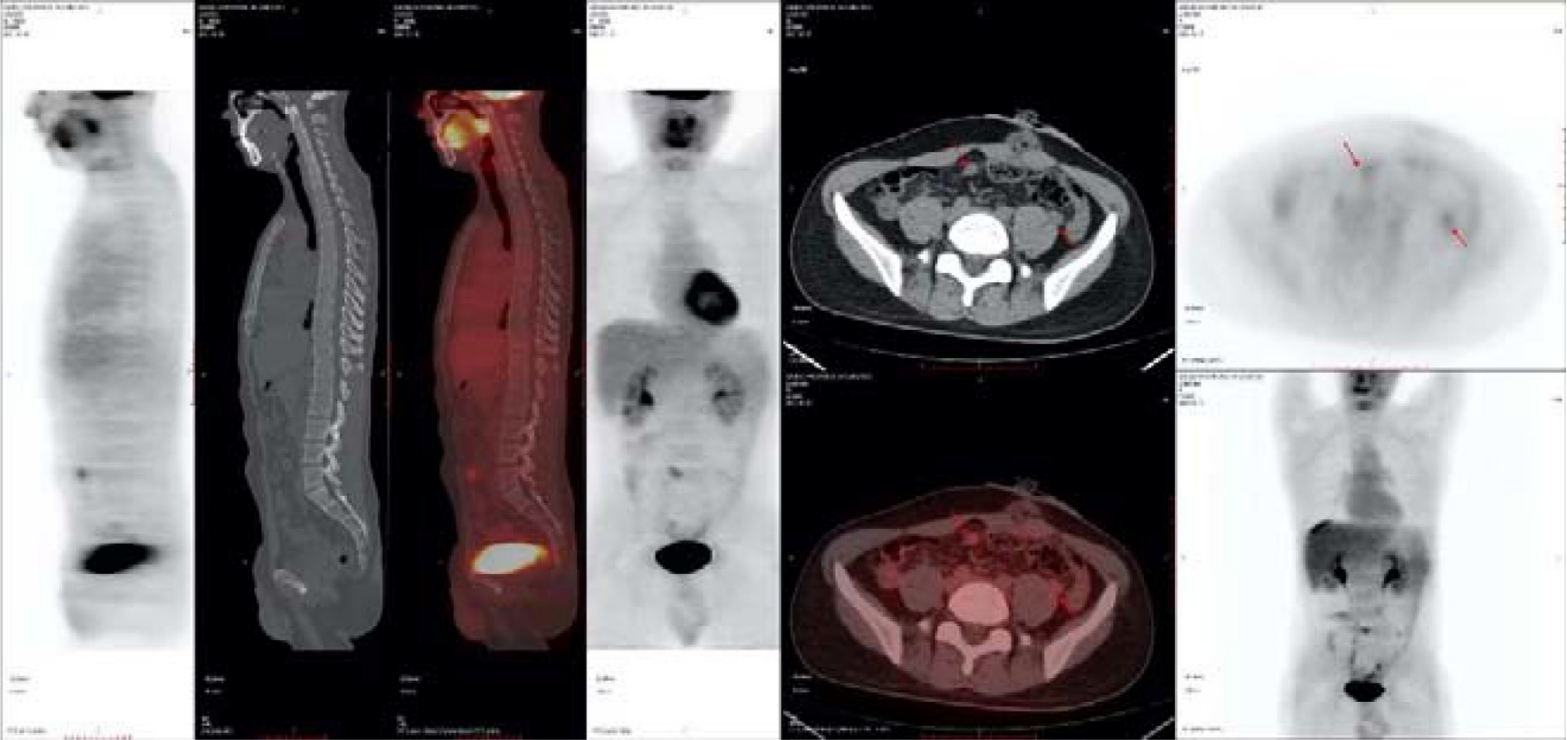
2021-1-12PET-CT.

After multiple rounds of discussions by the multidisciplinary team (MDT), the postoperative treatment for this patient is as follows:

After the surgery, intraperitoneal hyperthermic perfusion chemotherapy (Raltitrexed 5.3mg + Lobaplatin 53.4mg + Endostar 71.2mg) was performed. Subsequently, 11 cycles of Bev + mFOLFOX6 (L-OHP 150mg; CF 0.7g; 5-Fu 5g; Bev 340mg) treatment were given.PET-CT: After the surgery for left-sided colon cancer, multiple soft tissue nodular shadows were seen in the pelvic mesenteric space, with the largest one being approximately 12*11mm, accompanied by abnormal radioactive concentration, SUVmax 2.6 (**Figure 4**).

**Figure 5:**
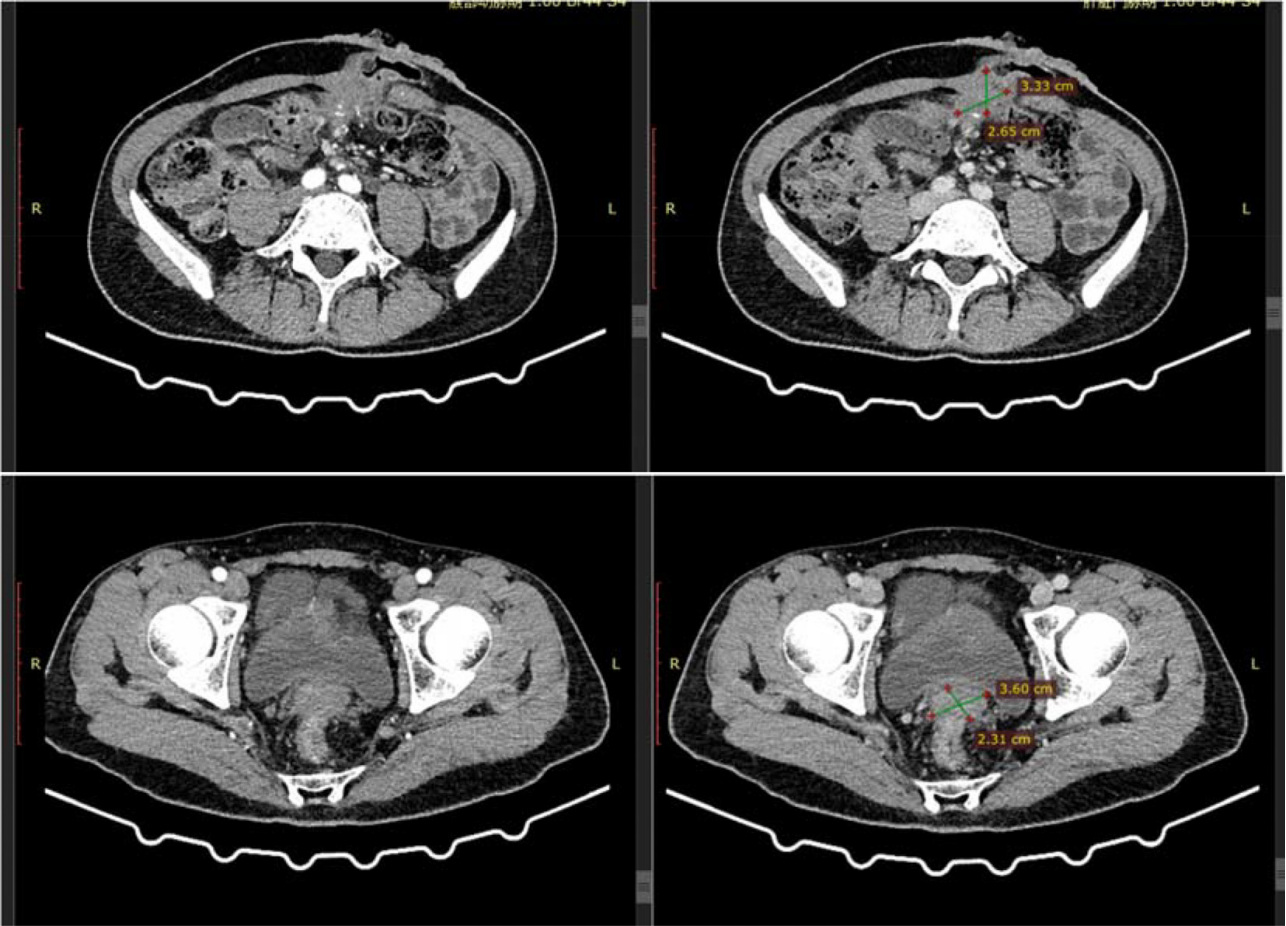
Abdominal CT in February 2023.

Subsequently, 10 cycles of BEV + FOLFIRI (CTP-11 320mg; CF 0.7g, 5-Fu 5g; Bev 340mg) chemotherapy combined with targeted therapy were administered. After a full abdominal MR on April 6, 2021, the efficacy evaluation showed a partial response (PR) of the tumor. A full abdominal MR was performed on July 2, 2021. The efficacy evaluation showed stable disease (SD). After the patient’s condition did not progress, 3 cycles of BEV + Capecitabine (Bev 340mg, Xeloda 124.6g) maintenance treatment were carried out. After a reexamination of MR on September 17, 2021, the efficacy evaluation showed disease progression (PD). The patient was advised to undergo anti-HER2 treatment, but due to the COVID-19 pandemic, the patient was unable to continue to participate in the subsequent treatment. On January 18, 2022, the abdominal CT evaluation showed PD. On March 15, 2022, the patient continued to come to the hospital for treatment and was given the first dose of Trastuzumab (544mg) + Pertuzumab (840mg), followed by 5 doses of Trastuzumab (408mg) + Pertuzumab (420mg) for a total of 6 cycles of treatment. On July 8, 2022, an abdominal CT was repeated to assess the partial response (PR). Subsequently, three cycles of trastuzumab combined with patuzumab were continuously administered. After the treatment, a reexamination of abdominal CT on October 9, 2022 showed PD in the efficacy evaluation. The treatment plan was adjusted to Regorafenib (160mg Po QD for 21 days) for 3 cycles. Abdominal CT showed SD in the efficacy evaluation. Regorafenib treatment was continued for 2 cycles. In the abdominal CT reexamination in February 2023, the efficacy evaluation was PD (**Figure 5**).

**Figure 6:**
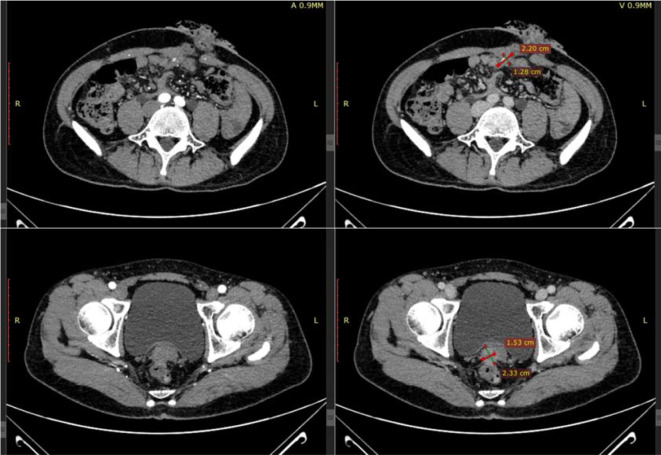
Three months after T-DXd treatment, abdominal CT in May 2023.

**Figure 7:**
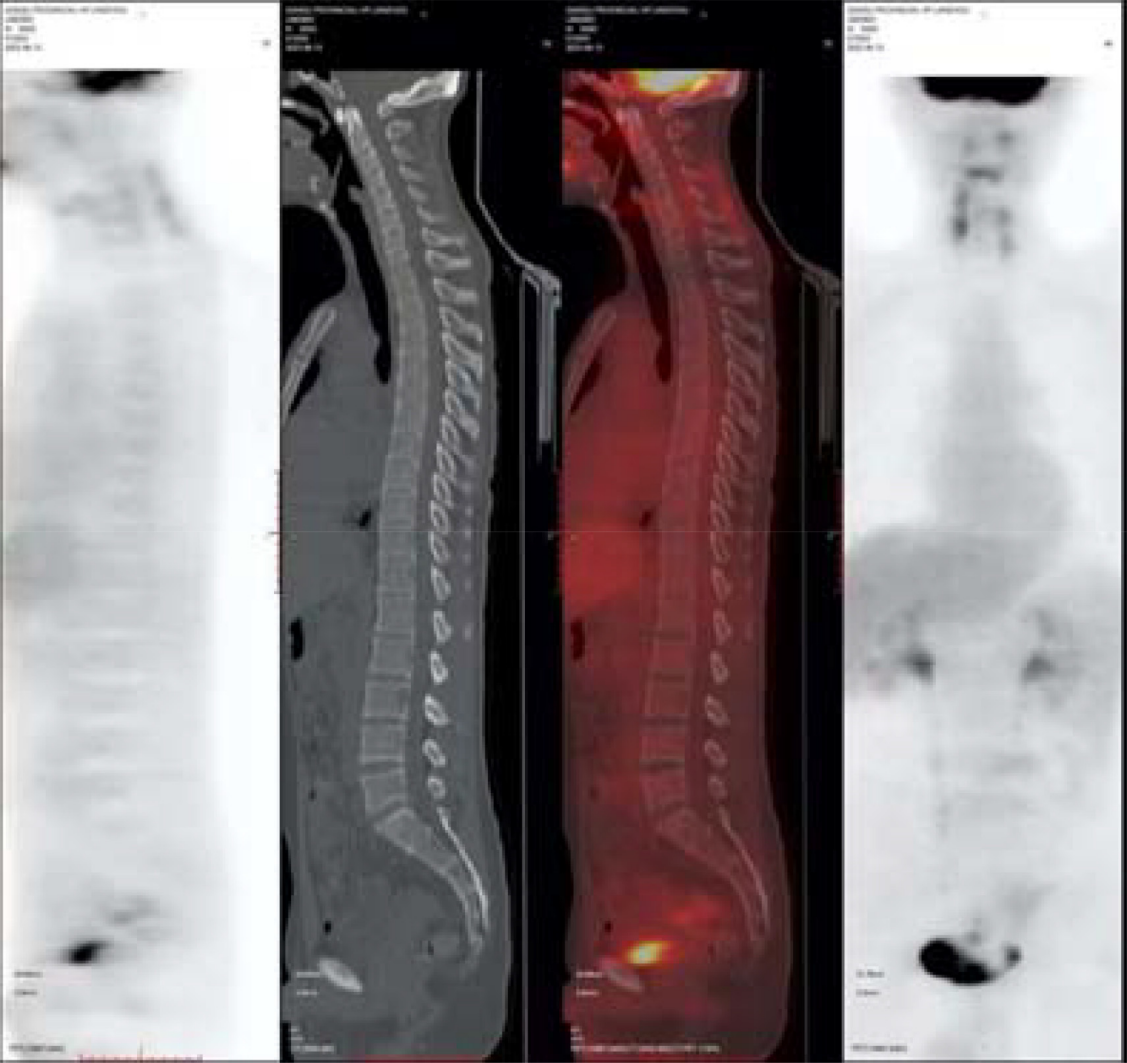
In June 2023, PET-CT.

After the patient’s disease progressed after anti-HER2 treatment, T-DXd (435.2mg) was given for 6 cycles within the following 3 months. In the abdominal CT reexamination in May 2023, the efficacy evaluation was PR (**Figure 6**). In June 2023, the PET-CT indicated: No distant metastasis (**Figure 7**).

**Figure 8:**
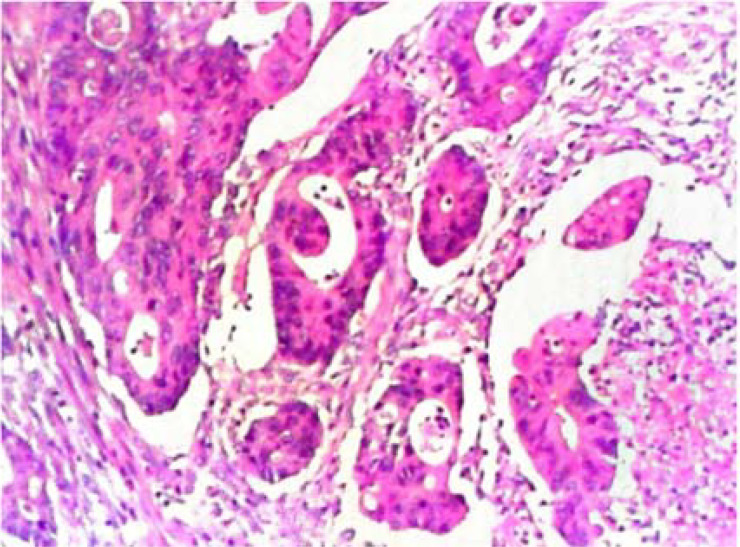
Postoperative pathological examination for the second time 10*20 HE.

**Figure 9:**
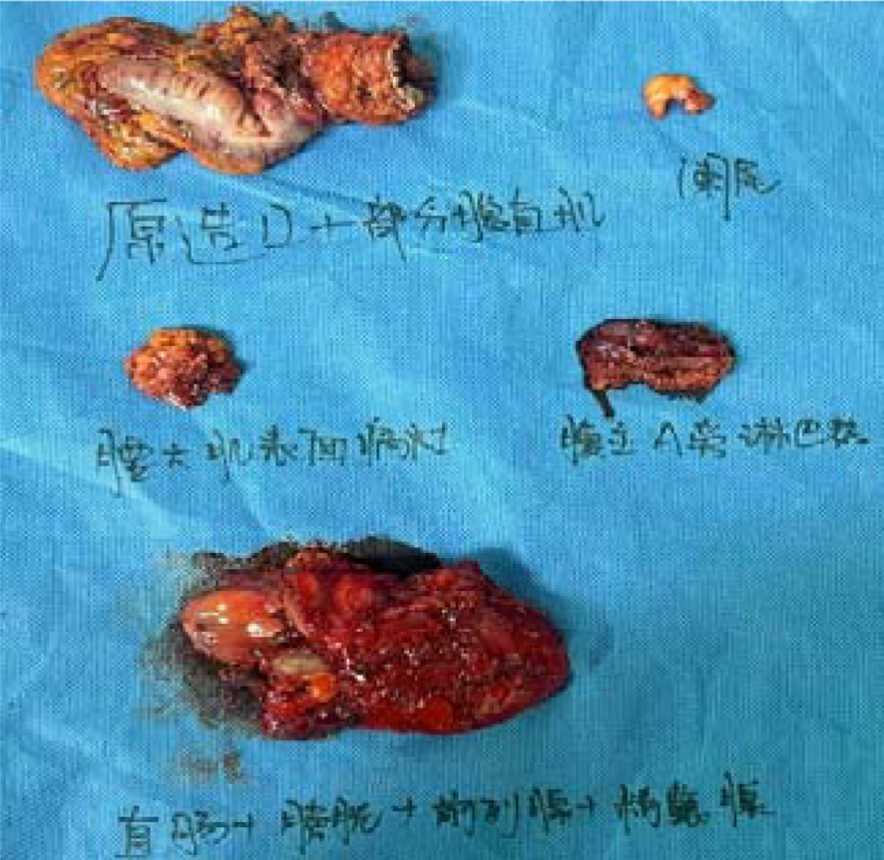
The gross specimen of the surgical resection.

After the anti-HER2 treatment with T-DXd was effective, on July 7, 2023, the operations of colostomy and resection of the metastasis beside the colostomy; partial rectus abdominis muscle resection; colostomy reversal; anterior resection of pelvic organs; para-aortic lymph node dissection; resection of the metastasis on the surface of the left psoas major muscle + partial psoas major muscle resection; appendectomy; ileal bladder substitution; ileostomy were performed. The postoperative pathological examination showed that a large amount of moderately differentiated tubular adenocarcinoma tissues were found in the rectal mucosa, the proper muscle layer, the perintestinal adipose tissue, the bladder wall mucosa, and the bladder wall muscle layer (**Figures 8** and **9**). According to the AICC/CAP pathological evaluation criteria after neoadjuvant chemotherapy: TRG: Grade 2 (Ryan classification: TRG Grade 1: Complete tumor regression, no residual tumor cells, only fibrous tissue is seen. TRG Grade 2: A small amount of residual tumor cells (single or small clusters of tumor cells). TRG Grade 3: A considerable amount of residual tumor cells, but less than fibrotic tissue. TRG Grade 4: Extensive residual tumor, no or a small amount of treatment response.). Metastatic carcinoma was found in the lesion on the surface of the psoas major muscle.

After 9 cycles of T-DXd (435.2mg) treatment after the surgery, the patient was evaluated as having No Evidence of Disease (NED). After comprehensive good physical condition, the ileostomy reversal operation was performed. There has been no recurrence or metastasis after the surgery. Currently, the patient is under regular follow-up.

## Discussion

In the diagnosis and treatment process of this case, through multidisciplinary collaboration and the joint implementation of multiple measures, multiple rounds of anti-HER2 treatment were given after conventional chemotherapy, and the NED state was achieved through surgery after the condition was controlled. Especially the MDT discussions before each adjustment of the treatment plan to formulate individualized diagnosis and treatment plans for the patient, which is also the key factor for the patient’s ultimate long-term survival.

Peritoneal metastasis of CRC refers to the metastasis of cancer cells from the primary lesion to the peritoneum through factors such as the shedding of cancer cells and the peritoneal microenvironment. Peritoneal metastasis is the third most common metastasis route after liver and lung in CRC, and the prognosis is usually poor (10). Its treatment mainly includes cytoreductive surgery combined with hyperthermic intraperitoneal chemotherapy (CRS + HIPEC), the combination of systemic chemotherapy and intraperitoneal chemotherapy. Commonly used drugs include 5-fluorouracil, oxaliplatin, etc., as well as targeted therapy, etc (11). The treatment of peritoneal metastasis of CRC is a complex process that requires a multidisciplinary comprehensive diagnosis and treatment. Throughout the entire diagnosis and treatment process of this case, we have always adhered to the multidisciplinary comprehensive treatment model in order to formulate the best diagnosis and treatment plan for the patient. With the in-depth study of its pathogenesis and the continuous development of treatment techniques, the prognosis of these patients has been further improved.

The hyperthermic intraperitoneal perfusion therapy (HIPEC) used in this case also has a unique role in preventing peritoneal metastasis of CRC. HIPEC technology is an individualized diagnosis and treatment method formulated after an individual assessment in the abdominal cavity. It can improve the overall survival period of patients, improve the overall quality of life of patients, and reduce tumor recurrence and peritoneal metastasis caused by advanced (pT4) CRC (12). In a study by Qiu Cen et al. on the treatment of allogeneic CRC peritoneal metastasis model with raltitrexed intraperitoneal hyperthermia, it was shown that HIPEC does not cause serious adverse reactions and can effectively reduce the spread of cancer cells (13).

HER 2 is a proto-oncogene, and its genomic amplification or mutation is the main mechanism for the occurrence of some malignant tumors. HER 2 genomic amplification is commonly seen in breast cancer (BC) and gastric cancer (GC), with incidences of approximately 25% and 15% respectively; while it is relatively rare in CRC, with an incidence of approximately < 5% (14). Research data indicate that the objective response rate of HER 2 -positive CRC patients treated with trastuzumab + lapatinib or trastuzumab + pertuzumab respectively is 30% - 38% (15, 16). HER2 is not only a therapeutic target for CRC, but also a resistance mechanism for epidermal growth factor receptor (EGFR) targeted therapy (such as cetuximab and panitumumab) (17, 18). In this case, the tumor progressed after 7 - month treatment with trastuzumab + pertuzumab, which may be due to the development of drug resistance during this period. On August 11, 2022, the US FDA officially approved the listing of ENHERTU (code DS8201, fam - trastuzumab deruxtecan - nxki, Chinese name: fam - trastuzumab deruxtecan for injection) for adult patients with inoperable and treated HER2 - positive non - small cell lung cancer (NSCLC). T-DXd is a conjugate drug of HER2 antibody and irinotecan-based chemotherapy drug, belonging to the ADC-type drug category (19). The drug-to-antibody ratio of T-DXd reaches 100%, which enables T-DXd to deliver more effective molecules to the targeted tumor cells. The payload of T-DXd has a short half-life, which can minimize off-target toxicity. In addition, the connection point is cleaved by the highly expressed lysosomal enzymes in tumor cells, which can make T-DXd more stable in the systemic circulation and maintain a mild systemic toxicity (16, 20–22). In the Phase 2 DESTINY-Breast01 trial, the patients in the experiment had previously received two or more HER2-based anti-HER2 regimens, and T-DXd showed an objective response rate of 60.9% and a median progression-free survival of 16.4 months (23). Jose Perez et al.’s study also mentioned that T-DXd is expected to treat HER2 - positive solid tumors, including CRC, non-small cell lung cancer, etc (24). In the DESTINY-PanTumor02 study, it is used to evaluate the efficacy and safety of T-DXd (5.4 mg/kg, once every 3 weeks) in patients with locally advanced or metastatic solid tumors with HER2 expression (IHC 3+ or 2+). Among all patients, the ORR was 37.1% (n = 99; [95% CI, 31.3, 43.2]). This experiment shows that T-DXd not only provides durable clinical benefits in a variety of HER2 - expressing solid tumors, but also the greatest benefit can be observed in the IHC 3+ patient population (8). In the field of CRC, the DESTINY-CRC01 study has brought a new therapeutic drug for HER2 - positive CRC. In this study, 86 patients were randomly divided into three cohorts according to the HER2 expression situation. The results showed that T-DXd not only showed excellent efficacy in the refractory patient population, but also its safety and tolerability were within the controllable range. In particular, T-DXd showed an ORR of 45.3% and a median OS of 15.5 months in cohort A, which provides a new treatment option for HER2 - positive mCRC patients (25). The DESTINY-CRC02 study confirmed that T-DXd is effective for HER2 + mCRC patients at doses of 5.4 and 6.4 mg/kg. The 5.4 mg/kg dose can achieve better results in this population (11). In the DS8201 - A - J101 study initiated in 2015, the researchers evaluated the safety and tolerability of T-DXd in advanced solid tumors with HER 2 - positive. The dose escalation part of the study determined 5.4 mg/kg or 6.4 mg/kg as the recommended dose (26). While T-DXd has shown excellent therapeutic effects, researchers have also conducted corresponding studies on the adverse reactions after its use. Common complications after the use of T-DXd: interstitial lung disease (including pneumonia), cardiotoxicity, neutropenia and left ventricular dysfunction. Other important common adverse reactions include nausea, fatigue, vomiting, hair loss, constipation, loss of appetite, anemia, diarrhea and thrombocytopenia, etc., but the overall side effects are minial (27). Therefore, the application of T-DXd is safe and reliable. Based on the above studies, we used T - DXd in the treatment process of this case, and after multiple rounds of treatment, the patient’s tumor significantly shrank and the pathological regression grade reached TRG: Grade 2.

## Conclusion

This article shares the diagnosis, treatment process and reflection of a patient with advanced malignant tumor of HER-2 positive descending colon cancer with peritoneal metastasis. After a clear diagnosis of the tumor in this case, the patient survived for a long time after surgery, HIPEC, systemic chemotherapy, and various targeted therapies, and achieved the No Evidence of Disease state after tumor cytoreductive surgery again.

In summary, the patient in this case achieved the NED state after multiple rounds of treatment and was able to survive for a long time, which is inseparably related to the implementation of MDT, relatively standardized systemic treatment, the application of anti-HER-2 drugs, the continuous progress of surgical techniques, and the patient’s perseverance.

## Data Availability

The original contributions presented in the study are included in the article/supplementary material. Further inquiries can be directed to the corresponding author.
